# Increased generosity under COVID-19 threat

**DOI:** 10.1038/s41598-022-08748-2

**Published:** 2022-03-31

**Authors:** Ariel Fridman, Rachel Gershon, Ayelet Gneezy

**Affiliations:** Rady School of Management, University of California, San Diego, San Diego, CA 92093 USA

**Keywords:** Psychology, Human behaviour

## Abstract

In the face of crises—wars, pandemics, and natural disasters—both increased selfishness and increased generosity may emerge. In this paper, we study the relationship between the presence of COVID-19 threat and generosity using a four-year longitudinal dataset (N = 696,942) capturing real donations made before and during the pandemic, as well as allocations from a 6-month dictator game study (N = 1003 participants) during the early months of the pandemic. Consistent with the notion of “catastrophe compassion” and contrary to some prior research showing a tendency toward self-interested behavior under threat, individuals across both datasets exhibited greater financial generosity when their county experienced COVID-19 threat. While we find that the presence of threat impacted individual giving, behavior was not sensitive to threat level. Our findings have significant societal implications and advance our understanding of economic and psychological theories of social preferences under threat.

## Introduction

During major crises, such as natural disasters, wars, and now the COVID-19 pandemic, two conflicting behaviors may emerge: increased selfishness or increased generosity. Selfishness is innate to our survival instincts^1,2^. Evidence suggests that when facing adverse circumstances, individuals’ may shift away from other-regarding practices^3^, arguably because fear and uncertainty resulting from increased risk perceptions^4^ drive self-preservation^5^. Along these lines, research finds that the presence of threat can decrease individuals’ willingness to engage in charitable activities and civic duties (e.g., paying taxes and reporting a crime^6^) and that generosity toward in-group and out-group members may decrease following a natural disaster^7^. Considering these findings, one might expect that individuals experiencing COVID-19 threat would, on average, behave more selfishly than those not experiencing threat. Indeed, at an early stage of the COVID-19 pandemic, survey data representing 35 countries showed that, despite government appeals, those who felt more threatened were more likely to engage in selfish stockpiling^8^, putting the health and well-being of others at risk^9^. Across the world, many of the widespread product shortages during the early months of the COVID-19 pandemic were triggered by consumers purchasing resources in excess of their actual need (e.g., hoarding toilet paper^10^ and masks^11^).

On the other hand, there is evidence suggesting that groups facing a common threat often demonstrate stronger social cohesion^12,13^ and more cooperative, communal behaviors^14–17^. As proposed by Jamil Zaki’s “catastrophe compassion” theory^18^, disasters may promote an increased sense of community and altruism^19^. Indeed, experiencing high (vs. low) stress can increase trust and sharing behaviors^20,21^. Perceived threat may also promote the expansion of social connections, as observed in monkeys in response to the environmental instability caused by Hurricane Maria^22^. Considering these findings, one might predict that experiencing and bearing witness to the devastating effects of the COVID-19 pandemic would promote generosity. Consistent with this proposition, a survey conducted by the Lilly Family School of Philanthropy^23^ during the COVID-19 pandemic found that nearly half of respondents supported their communities in a variety of ways, for example by continuing to pay individuals and businesses for services that could not be rendered^24^.

Interestingly, research suggests that the impact of threat could go either way—increasing or decreasing generosity. For example, when primed with resource scarcity, individuals became more competitive, causing some to become more selfish while others to exhibit greater generosity, depending on the context (e.g., behavior observability)^25^. Additional research finds evidence that communities experiencing disasters could simultaneously undergo positive and negative behavioral change^26^.

We examine the relationship between COVID-19 threat and generosity using two independent longitudinal datasets. The first dataset, provided by Charity Navigator (CN), the world’s largest independent charity evaluator, consisted of actual charitable-giving data spanning July 2016 through December 2020 (N = 696,942 donations). For each donation, the data included the donation amount, the charities benefited, each charity’s assigned category (e.g., environment and human services), and the donor’s location. In addition, CN assigned each donor a unique identifier, which allowed us to observe within-person differences in donation behavior in both the presence and absence of COVID-19 threat.

The second dataset, which sheds light on the relationship between COVID-19 threat and generosity in a more controlled setting, consisted of individuals’ (N = 1003 U.S. participants) allocations from an incentivized dictator game. In the dictator game, one player (the *dictator*) receives $10 and makes a unilateral decision on how to divide it between themselves and another, typically unknown, individual^27,28^. Rather than maximizing their own financial payoff (i.e., allocating $0), experimental evidence shows that “dictators” often choose to give some of their money to recipients^29–31^. In our study, participants played an incentive-compatible dictator game monthly, from March to August 2020. Importantly, at the start of this period, COVID-19 threat was only present in 10% of participants’ counties (see SI Appendix, Table S1), allowing us to observe their behavior when threat was first introduced in most counties. Notably, while the dictator game has previously been used to capture generosity at a single point in time, there are few cases of its use in a longitudinal setting^7,32^. Finally, our research is unique in the use of longitudinal dictator game data jointly with longitudinal archival data to show convergent evidence of changes in giving behavior.

While our observational datasets do not lend themselves to causal claims, it is reasonable to infer that the presence of threat would increase generosity^12,14–16,18–24^, while reverse causality is highly unlikely. See “Discussion” section for a more detailed explanation.

Our large-scale longitudinal datasets provide real-world evidence that people exhibited greater generosity during a time where some theories and experts predicted the opposite due to the economic downturn associated with the pandemic. While our analyses consider various levels of threat, we found that only the presence (vs. absence) of threat was associated with greater generosity. Our findings contribute to economic and psychological theories of social preferences, suggesting that people come together in the presence of a shared threat and demonstrate a willingness to support others, despite the uncertainty surrounding their own health and financial well-being.

## Results

Across both datasets, we observe increased generosity in the presence of COVID-19 threat in participants’ geographic location.

Our analyses controlled for potential confounds at the national level (e.g., stimulus payouts and the Black Lives Matter movement) by including date fixed effects in all regressions. See “Materials and methods” section for details.

### Charity Navigator

**Figure 1:**
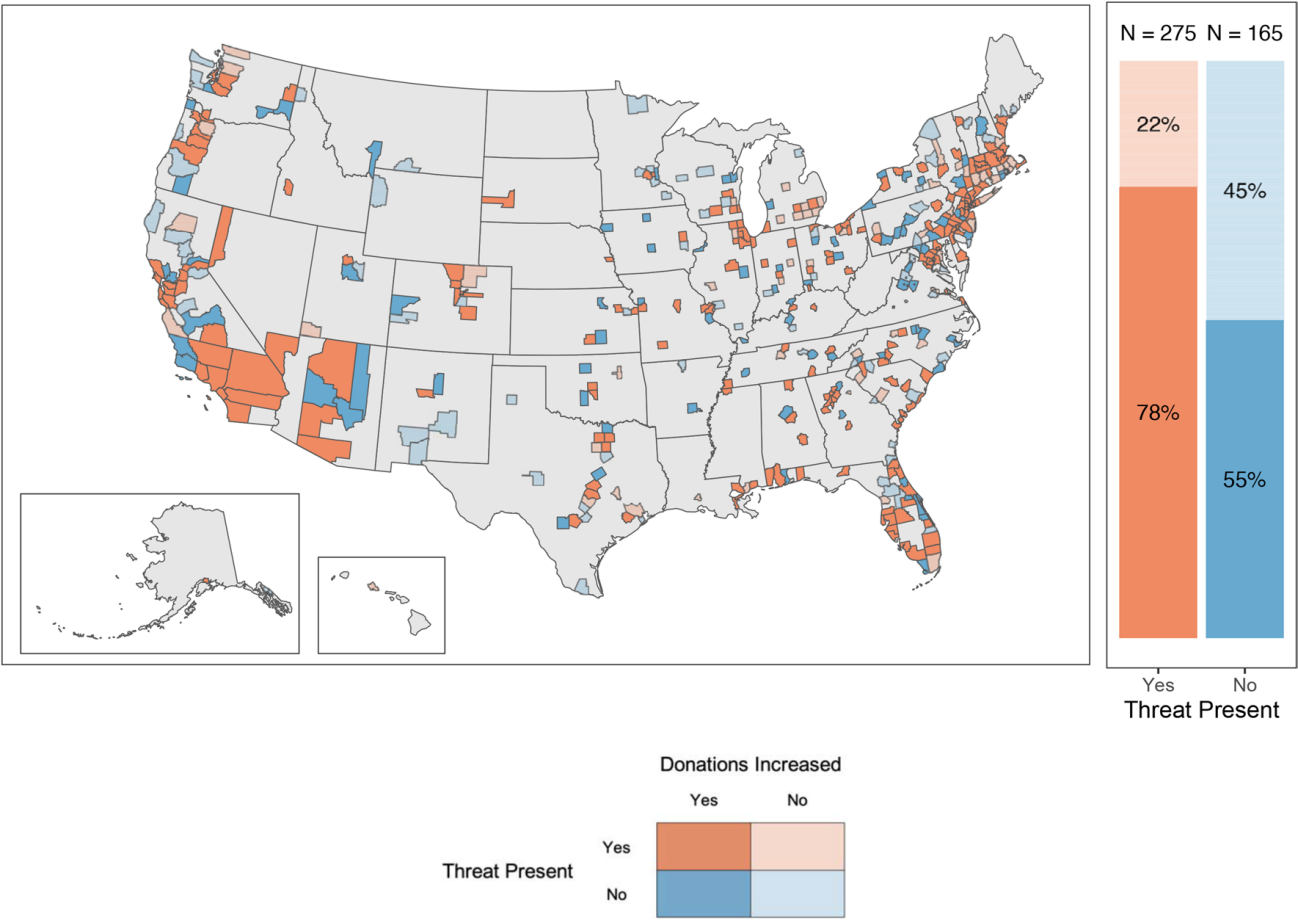
Counties by COVID-19 threat and donation changes—March 2019 versus March 2020. Orange [blue] represents the presence [absence] of threat in March 2020. Darker [lighter] shades indicate an increase [decrease] in giving across all charity categories relative to March 2019. The map shows U.S. counties with inset maps for counties in Alaska and Hawaii. The chart on the right shows the proportion of counties in each “threat present” and “donations increased” group.

We first analyzed the data with a relatively simplified approach by treating COVID-19 threat as a binary variable—whether any COVID-19-related deaths (vs. no COVID-19 deaths) occurred in each calendar month. In this analysis, we compared the proportion of counties that increased their overall donation amount as a function of whether the county had experienced COVID-19 threat. Compared with March 2019, 78% of counties that experienced threat increased the total amount donated in March 2020. Of the counties that did not face threat, 55% increased giving (χ^2^(1, N = 440) = 24.75, *P* < .001; see Fig. 1). We found similar results for the comparison between April 2019 and April 2020 (see SI Appendix, Fig. S1). After April 2020, only 33 counties or fewer did not face threat, though results were directionally the same for all months except August and October. While we treat counties with no COVID-19 deaths as “no threat”, it is possible that some people in these counties still experienced COVID-19 threat, suggesting that our analysis is conservative. These results present initial model-free evidence for our main finding—that a greater proportion of counties experiencing threat increased giving, compared with those facing no threat.

To examine the relationship between COVID-19 threat level and county-level donation amount, we ran a regression analysis using county-level data aggregated by month-year. We captured the COVID-19 threat level using a 7-day average of daily new deaths per million in each county, which we averaged over all days in each month and binned into four categories by quantiles (no, low, medium, or high threat; log transformation generated similar results, see SI Appendix, Tables S2 and S3). We binned the threat variable to allow for non-linear relationships between threat and charitable giving. We regressed log-transformed aggregated giving amounts on threat level and included county and month-year fixed effects. This analysis showed that, overall, giving through CN’s platform increased across all threat levels compared with no threat. On average, county-level giving increased 31.6% under low threat (SE = 0.06, *t* = 4.94, *P* < .001), 28.5% under medium threat (SE = 0.07, *t* = 3.86, *P* < .001), and 32.9% under high threat (SE = 0.05, *t* = 6.10, *P* < .001), relative to periods of no threat in the county. All pairwise comparisons across low, medium, and high threat levels were non-significant (*P* > .32), suggesting insensitivity to threat magnitude.

To examine whether our findings held within individuals (i.e., among repeat donors), we analyzed individual-level data, which allows us to rule out potential selection bias (i.e., changes in donor characteristics before and during the COVID-19 pandemic). Of those who donated in 2020, 32% were repeat donors, meaning they made donations through the platform more than once. We captured the level of COVID-19 threat using the same seven-day lagged moving average of daily new deaths per million (without month-level averaging), considering “no threat” as our baseline. A regression of log-transformed giving amounts on threat level, including individual and date fixed effects, revealed that repeat donors’ giving increased significantly across all charity categories by 3.4% under high threat (SE = 0.02, *t* = 2.11, *P* = .040), and non-significantly by 1.3% under medium threat (*P* = .348), and 2.8% under low threat (*P* = .063). All pairwise comparisons across low, medium, or high threat levels were non-significant (*P* > .07).

**Figure 2:**
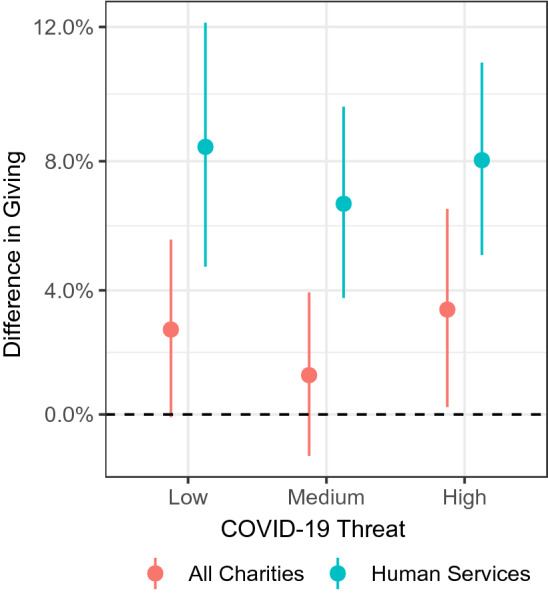
Charity Navigator donations. The vertical axis captures the difference between giving under threat relative to no threat (dashed line). The horizontal axis indicates threat levels in each participant’s county at the time of the donation. Points and error bars represent regression coefficient estimates and 95% confidence intervals, respectively. Note the “All charities” category includes human services charities.

Although this effect is smaller than the effect we observe with the county-level model, including interaction terms with the charity category revealed a highly significant increase in donations to human services charities—organizations that provide direct services to those in need (see SI Appendix, Table S4). While one might expect an increase in donations to health charities, this category includes organizations such as Planned Parenthood and Cure Alzheimer's Fund, which are not directly related to the COVID-19 pandemic. In contrast, human services charities include food banks and homeless services. Among repeat donors, donations to human services charities increased by 8.4% under low threat (SE = 0.02, *t* = 4.48, *P* < .001), 6.7% under medium threat (SE = 0.02, *t* = 4.47, *P* < .001), and 8.0% under high threat (SE = 0.01, *t* = 5.39, *P* < .001), relative to periods of no threat in the donor’s county (Fig. 2). All pairwise comparisons across low, medium, or high threat levels were non-significant (*P* > .29). Collapsing all charity categories except human services revealed no significant difference in donations in response to the presence of COVID-19 threat (*P*s > .55 for low, medium, and high threat). Notably, our analysis revealed a significant interaction of COVID-19 threat with human services charities (*P*s < .001), indicating that under COVID-19 threat in one’s county, repeat donors were significantly more likely to donate to human services charities than to other types of charities (see SI Appendix, Table S4). Together, these findings suggest giving to human services charities increased under threat but not at the expense of donations to other charity categories.

In an additional analysis, we found that the observed county-level increases in giving did not vary by county-level median household income (see SI Appendix, Table S5 for results).

### Dictator game

**Figure 3:**
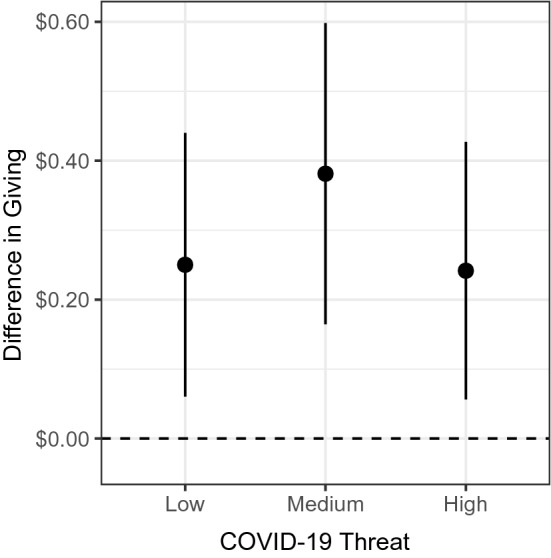
Dictator game allocations. The vertical axis captures the difference between giving under threat relative to no threat (dashed line). The horizontal axis indicates threat levels in each participant’s county at the time of the survey. Points and error bars represent regression coefficient estimates and 95% confidence intervals, respectively.

An analysis of our six-wave longitudinal dictator-game data also showed that participants gave significantly more under the presence of COVID-19 threat. We used the same threat measure and individual-level regression model as the CN analysis. Our outcome measure was participants’ allocation decisions, and we used wave fixed effects instead of date fixed effects. We found that within-person giving increased by approximately $0.25 (8.6%) under low threat (SE = 0.10, *t* = 2.58, *P* = .013), $0.38 (13.1%) under medium threat (SE = 0.11, *t* = 3.45, *P* = .001), and $0.24 (8.3%) under high threat (SE = 0.09, *t* = 2.56, *P* = .014), relative to periods of no threat in the participant’s county (Fig. 3 and SI Appendix, Table S6). Percentages were calculated relative to a mean allocation of $2.92. All pairwise comparisons across low, medium, and high threat levels were non-significant (*P* > .10). Our analysis further revealed a significant interaction between allocation amount and gender, indicating women gave more than men under threat (collapsed across low, medium, and high threat levels, *P* = .033). We found no interaction between allocation amount and age. Unlike some other COVID-19-related behaviors^33–35^, we found no difference in giving patterns based on political affiliation.

## Discussion

Researchers have long argued that experiencing threat influences social preferences^18^, but offer different predictions for how. While some propose that those facing threat will become more generous, others predict increased selfishness. Leveraging data from a naturally-occurring state of emergency—the COVID-19 pandemic—we investigated the relationship between local COVID-19 threat and generosity.

The present work offers several important findings. First, analyses of both datasets show that individuals exhibited greater financial generosity under COVID-19 threat. The CN data indicates contributions were directed primarily toward charities in the human services category—organizations that help mitigate the effects of COVID-19. Second, although we examined local COVID-19 threat, increased generosity often emerged in support of non-local organizations (CN data) or unidentified individuals (dictator game data). CN’s data also show that the increase in donations was exhibited by both repeat and new donors, suggesting an overall increase in giving, as opposed to a mere allocation shift. We note that although both datasets demonstrate an association between the presence of threat and generosity, we found insensitivity to the level of threat. Although merely speculative, this pattern is consistent with research demonstrating scope insensitivity in emotionally charged settings^36–38^. Finally, the increased generosity observed across both datasets is particularly intriguing in light of expert predictions, based on historical data, that the economic downturn caused by the pandemic would lead to reduced giving^39^, and the fact that a record-high majority of Americans reported a worsening financial situation during the same period^40^. Prior work suggests that when people experience such financial scarcity, they may engage in extreme, even immoral, behaviors to acquire financial wealth^41,42^. Yet analyses of both our datasets clearly shows that in this particular circumstance, individuals were, on average, more willing to part with their financial resources.

From a methodological perspective, our results lend credibility to the dictator game as a reliable measure of real-world generosity^43,44^, because our dictator-game findings are consistent with CN’s field data. Thus, this work adds to a growing discussion in the literature regarding the validity of lab findings^45,46^. The present research is also unique with respect to the longitudinal nature of our data, which, as noted in a recent call for the integration of such data^47^, is largely absent from behavioral research, and particularly rare in the context of major crises^22^.

Although both datasets show that the presence of local COVID-19 threat is associated with increased generosity, our observational data does not necessarily lend itself to causal claims. With that in mind, we believe it is reasonable to infer that the presence of threat would influence generosity^12,14–16,18–24^; it is improbable, however, that the increase in generosity would trigger an increase in local COVID-19 threat. While we cannot rule out the possibility that there could be another variable that influences both factors to produce the observed correlation, our analyses and contemplation do not point to any likely candidates. However, it is possible that the presence of COVID-19 threat influences other variables (e.g., local lockdowns, media attention, etc.), which in turn lead to an increase in generosity. Nonetheless, we cautiously conclude that the presence of local COVID-19 threat led, either directly or indirectly, to an increase in generosity.

We are unable to discern what mechanism underlies the observed increase in generosity. Individuals may have been motivated to give more when experiencing threat as a result of increased feelings of sympathy^48^, a desire to regain a sense of agency and self-efficacy^49,50^, mortality salience^51,52^, or simply to experience positive emotions (e.g., warm glow^53,54^) during a stressful period. It is also possible our findings reflect self-interested behavior if people increased their donations to benefit themselves, thinking that doing so would help to mitigate the negative effects of COVID-19 in general. However, it is unlikely that this motive explains the dictator game findings, where giving benefits a randomly chosen anonymous individual. Based on our CN data, it also seems likely individuals donated to honor those who passed away during the pandemic or were otherwise affected (e.g., sick, lost a loved one, etc.). Indeed, a closer look at the CN data shows that the proportion of donations made “in memory of” someone was significantly greater in 2020 than every prior year, although this motivation is likely less salient in our longitudinal dictator-game findings.

Since our CN data represents individuals with financial means to give, we cannot rule out the possibility that our findings apply to a subset of the population. However, we found similar results using the dictator-game data, which likely represents a less affluent demographic^55^, and an analysis comparing changes in giving on CN by county-level median household income also found no significant differences.

While our work focuses on the early period of the COVID-19 pandemic, additional research is needed to understand the dynamics of the relationship between threat and generosity in the longer term, as well as once the crisis has ended (e.g., see^7^ for a dictator-game study before and after Cyclone Pam in 2015).

Finally, while our results show an increase in financial generosity under COVID-19 threat, it is possible that the pandemic also resulted in selfish behavior unobserved in our data (e.g., hoarding resources). Future research can investigate when, why, and for whom these divergent reactions may occur.

This work adds to our understanding of human behavior during times of crisis. Amidst the uncertainty, fear, and tragedy of the pandemic, we find a silver lining: people became more financially generous toward others in the presence of COVID-19 threat.

## Materials and methods

### Human subject protections

The longitudinal dictator game received ethical approval from the UC San Diego Institutional Review Board (Project #191273XX) and was carried out in accordance with the guidelines and regulations for a human research study. Informed consent was obtained for all participants. No personal identifying information was collected to ensure participants’ privacy.

### COVID-19 threat

To capture COVID-19 threat, we used the Center for Systems Science and Engineering at Johns Hopkins University (https://github.com/CSSEGISandData)^56^ time-series data of daily new deaths at the county level, which also includes U.S. Census Bureau population data by county. Our measure represented a seven-day lagged moving average of daily new deaths per million, which we binned into four categories based on population-weighted quantiles, taken over the entire timespan of the COVID-19 data in 2020: no deaths (0), bottom third (1), middle third (2), and top third (3). Consistent with prior work^57^, we used deaths rather than confirmed cases for the threat measure, because it is more consistent over time, as changes in confirmed cases may capture changes in testing availability, particularly at the beginning of the pandemic^58,59^.

To merge participant data from the dictator game and donor data from CN with our COVID-19 threat measure, we mapped participants’ zip codes to the corresponding county using the U.S. Department of Housing and Urban Development (https://www.huduser.gov/portal/datasets/usps_crosswalk.html) data^60^.

County-level median household income data was obtained from the U.S. Department of Agriculture’s Economic Research Service (https://www.ers.usda.gov/data-products/county-level-data-sets/)^61^.

#### Charity Navigator

To measure changes in generosity over time, we analyzed a dataset of donations made through CN. For each donation, the data specified the following: date, donation amount, charity name, a unique donor identifier (anonymized email address), and donor zip code. After excluding incomplete observations (0.9%; see SI Appendix, Text S1), we were left with 696,942 donations.

#### Dictator game

We recruited a representative panel of U.S. residents (see SI Appendix, Text S2) on Amazon’s Mechanical Turk platform to respond to monthly survey waves from March to August 2020. The sample consisted of 1003 unique participants. The first wave included 998 participants, ranging between 605 and 755 thereafter (5 participants did not respond to the dictator game in the first wave, but did so on subsequent waves; for attrition details, see SI Appendix, Table S1). After excluding incomplete observations due to missing county information (0.01%), we were left with 4272 observations.

Participants played the dictator game^31,62^ on all six survey waves and were always assigned to the dictator role. Participants allocated an amount between $0 and $10 to a randomly selected participant. To incentivize responses, we informed participants that, in each wave, one randomly selected participant would receive a $10 bonus, which would be split between them and another randomly selected participant according to their decision. Our survey also included demographic questions capturing age, gender, and political party affiliation.

### Regression models

For our primary analysis, we examined the relationship between COVID-19 threat and giving using multiple regression.

#### Charity Navigator

In our county-level specification, we aggregated the data to the month-year level by county and estimated the following model:

$$log\;(y_{cmy} ) = \mathop \sum \limits_{s = 1}^{3} \left( {\beta_{s} 1_{{threat_{cmy} = s}} } \right) + \alpha_{c} + \alpha_{my} + \varepsilon_{cmy} ,$$

where $y_{cmy}$ is the sum of the amount donated over all individuals in county *c* in month *m* in year *y*, $\alpha_{c}$ are county-level fixed effects, and $\alpha_{my}$ are month-year fixed effects. $threat_{cmy}$ is the average threat level in county *c* in month *m* in year *y*, where *s* indexes the threat level and coefficients $\beta_{s}$ measure the effect of COVID-19 threat on giving, relative to no threat. We log-transformed the amount donated because it was right-skewed. To test whether the effect of threat varied by median household income, we ran a similar specification, including interaction terms for median household income and $threat_{cmy}$. Standard errors were clustered by state and the regression was weighted by county population.

In our individual-level specification, we exploited within-person variation in giving over time, estimating the following model:

$$log\;(y_{id} ) = \mathop \sum \limits_{s = 1}^{3} \left( {\beta_{s} 1_{{threat_{{c_{i} d}} = s}} } \right) + \alpha_{i} + \alpha_{d} + \varepsilon_{id} ,$$

where $y_{id}$ is the amount donated by individual *i* on date *d*, $\alpha_{i}$ are individual-level fixed effects, and $\alpha_{d}$ are date fixed effects. $threat_{{c_{i} d}}$ is the threat level in county *c* of individual *i* on date *d*. To estimate the effect of COVID-19 threat on donations for each category of charities, we ran a similar specification, including charity-category dummy variables and interaction terms for $threat_{{c_{i} d}}$ and each charity category. Standard errors for both regressions were clustered by individual and state.

#### Dictator game

For the dictator-game analysis, we used a similar approach and estimated the following model:

$$y_{iw} = \mathop \sum \limits_{s = 1}^{3} \left( {\beta_{s} 1_{{threat_{{c_{i} w}} = s}} } \right) + \alpha_{i} + \alpha_{w} + \varepsilon_{iw} ,$$

where $y_{iw}$ is the amount given by individual *i* on survey wave *w*, $\alpha_{i}$ are individual-level fixed effects, and $\alpha_{w }$ are survey-wave fixed effects. $threat_{{c_{i} w}}$ is the threat level in county *c* of individual *i* on wave *w*, where *s* indexes the threat level and coefficients $\beta_{s}$ measure the effect of COVID-19 threat on giving, relative to no threat. Standard errors were clustered by individual and state.

See SI Appendix, Text S3 for additional methodological information.

## Supplementary Information

Supplementary Information.

## Data Availability

Our materials, anonymized behavioral data, and additional analyses, including robustness checks, are available on OSF (https://osf.io/2rykb/).
