# Supplementary material for: Increased generosity under COVID-19 threat
Source: Sci Rep. 2022 Mar 31;12:4886. doi: 10.1038/s41598-022-08748-2 (PMC8971415; doi:10.1038/s41598-022-08748-2)
Supplement: Supplementary file 1 — Supplementary Information. [file 41598_2022_8748_MOESM1_ESM.docx]

**Supplementary Information for**

Increased Generosity under COVID-19 Threat.

Ariel Fridman^1^*

Rachel Gershon^1^

Ayelet Gneezy^1^

^1^Rady School of Management, University of California, San Diego, San Diego, CA, 92093, USA

*Corresponding Author

**Email:**  [afridman@ucsd.edu](mailto:afridman@ucsd.edu)

**This PDF file includes:**

Supplementary text S1 to S3

Figure S1

Tables S1 to S6

SI References

Supplementary Information Text S1

Additional Information for Charity Navigator.

The Charity Navigator’s dataset used in this analysis spans from July 22, 2016, through December 10, 2020; the dictator-game was administered monthly from March 16 to August 16, 2020.

We excluded incomplete entries (i.e., missing email addresses, zip codes not matched to counties, or counties missing COVID-19 data) from our CN dataset, accounting for 0.9% of observations. We included donations that were refunded in our analysis (about 0.14% of donations), because almost all donations from September 21, 2020, onward were not yet determined to be refunded or not.

Supplementary Information Text S2

Additional Information for Dictator Game.

To incentivize completion of all survey waves, we informed participants in Wave 1 that their payment would increase for subsequent surveys and that those completing the first three waves would enter a $100 raffle. Participants were paid $0.30 for wave 1, $0.40 for wave 2, $0.60 for waves 3 and 4, $1.00 for wave 5, and $1.20 for wave 6. The median survey completion time was 5.5 minutes.

Our panel represented the broad and diverse population of the U.S. The first-wave sample included participants from all 50 states (except Wyoming) and Washington D.C., with an age range of 18 to 82 years old (mean = 38.48, median = 35). Approximately half of our participants (53%) identified as male, 46% as female, and .6% as other. The racial makeup in our sample was 80% White, 9% Asian, 6% Black or African American, 4% multiple racial or ethnic identities, and 1% other. Relative to the U.S. Census (2019) (1) estimates, our sample over-represents White and Asian individuals and under-represents Black or African American individuals and other racial groups.

Supplementary Information Text S3

Additional Methodological Information.

All analyses were conducted using R (version 4.0.2), and regressions were run using the package “fixest” (version 0.6.0).

For the measure of COVID-19 threat, we determined category cutoffs using county-level daily data spanning January 22, 2020 (the earliest available) to December 31, 2020, for every county in the U.S. We classified observations with a value of 0 on the COVID-19-threat measure as no threat, and classified the remaining observations as low, medium, or high threat based on whether they fell in the bottom, middle, or top third (population-weighted), respectively.

In both the dictator game and CN dataset, we mapped each participant’s zip code to the corresponding county, allowing us to merge each dataset with the COVID-19-threat data. If a zip code matched multiple counties, we matched it to the county with the larger population.

Figure S1. April 2019 vs. 2020 County Map.

Similar to the results shown for March in Fig. 1, a greater proportion of counties facing threat increased giving, compared with those facing no threat. Specifically, compared with April 2019, 84% of counties experiencing threat increased the total amount donated in April 2020. Of the counties that did not face a threat, 63% increased giving (χ2(1, N = 464) = 14.13, P < .001).

Fig. S1. Orange [blue] represents the presence [absence] of threat in April 2020. Darker [lighter] shades indicate an increase [decrease] in giving across all charity categories relative to April 2019. The main map shows U.S. counties with inset maps for counties in Alaska and Hawaii. The chart on the right shows the proportion of counties in each “threat present” and “donations increased” group.

Table S1. Dictator-Game Attrition.

To rule out differential attrition, we tested whether the composition of our sample (e.g., age, gender, and political party) changed over time. Specifically, we tested whether participants who responded to waves 2–6 were significantly different at baseline (wave 1) from the entire sample in wave 1. The only significant change detected (Ps < .05) was with respect to participants’ age, though the differences were small—the average age was 38.5 in the first wave, and among participants who responded to subsequent waves, the baseline average age ranged from 39.9 and 40.8. We found no other systematic pattern of attrition among participants.

| **Variable** | **Wave Participants** | **Wave 1 Mean (SD)** | **N** |
| --- | --- | --- | --- |
| Age | 1 | 38.48 (12.21) | 997 |
|  | 2 | 39.93 (12.54)* | 762 |
|  | 3 | 40.59 (12.49)*** | 654 |
|  | 4 | 40.80 (12.43)*** | 608 |
|  | 5 | 40.14 (12.47)** | 651 |
|  | 6 | 40.33 (12.39)** | 666 |
| Gender (female) | 1 | 46.19% | 998 |
|  | 2 | 46.19% | 762 |
|  | 3 | 46.02% | 654 |
|  | 4 | 45.72% | 608 |
|  | 5 | 45.86% | 652 |
|  | 6 | 47.08% | 667 |
| Political Party (1 = strongly Republican; 6 = strongly Democratic) | 1 | 3.98 (1.49) | 998 |
|  | 2 | 3.95 (1.50) | 762 |
|  | 3 | 4.00 (1.50) | 654 |
|  | 4 | 3.95 (1.52) | 608 |
|  | 5 | 3.97 (1.52) | 652 |
|  | 6 | 4.00 (1.49) | 667 |
| Dictator Game Allocations ($0-$10) | 1 | 2.97 (2.54) | 998 |
|  | 2 | 2.97 (2.44) | 757 |
|  | 3 | 2.90 (2.49) | 649 |
|  | 4 | 2.91 (2.47) | 604 |
|  | 5 | 2.91 (2.47) | 649 |
|  | 6 | 2.95 (2.46) | 665 |
| No COVID-19 Threat | 1 | 89.05% | 986 |
|  | 2 | 89.52% | 754 |
|  | 3 | 89.01% | 646 |
|  | 4 | 90.37% | 602 |
|  | 5 | 90.08% | 645 |
|  | 6 | 90.47% | 661 |

Table S1. Table shows the baseline (wave 1) characteristics of respondents to each survey wave. For gender, only the proportion of females is shown; participants identifying as “another gender” constituted around 0.65% of the sample in each wave. For all variables, we tested whether participants who responded to waves 2-6 were significantly different at baseline (wave 1) from the full sample at baseline (chi-squared test for gender and threat, t-test for all others). The number of observations across variables vary somewhat because respondents were allowed to skip questions. Significance codes: *** P < .001, ** P < .01, * P < .05.

**Table S2. Charity Navigator Regression Table – Logged Threat Level.**

|  | **County-Month Aggregation** | **Individual-Level** | **Individual-Level** |
| --- | --- | --- | --- |
|  |  |  |  |
| Threat Level (logged) | 0.0396* (0.0196) | 0.0096 (0.0058) |  |
| Category: Arts, Culture, Humanities |  |  | -0.1438*** (0.0127) |
| Category: Community Development |  |  | 0.1892*** (0.0076) |
| Category: Education |  |  | 0.0182* (0.0082) |
| Category: Environment |  |  | 0.0500*** (0.0047) |
| Category: Health |  |  | 0.1159*** (0.0047) |
| Category: Human and Civil Rights |  |  | 0.0694*** (0.0067) |
| Category: Human Services |  |  | 0.1378*** (0.0051) |
| Category: International |  |  | 0.1927*** (0.0053) |
| Category: Religion |  |  | 0.0707*** (0.0203) |
| Category: Research and Public Policy |  |  | -0.6576*** (0.0218) |
| Threat Level (logged) x Category: Animals |  |  | -0.0085 (0.0077) |
| Threat Level (logged) x Category: Arts, Culture, Humanities |  |  | 0.0146 (0.0115) |
| Threat Level (logged) x Category: Community Development |  |  | 0.0050 (0.0104) |
| Threat Level (logged) x Category: Education |  |  | 0.0152 (0.0109) |
| Threat Level (logged) x Category: Environment |  |  | -0.0107 (0.0093) |
| Threat Level (logged) x Category: Health |  |  | -0.0142† (0.0083) |
| Threat Level (logged) x Category: Human and Civil Rights |  |  | 0.0120 (0.0078) |
| Threat Level (logged) x Category: Human Services |  |  | 0.0339*** (0.0068) |
| Threat Level (logged) x Category: International |  |  | -0.0071 (0.0064) |
| Threat Level (logged) x Category: Religion |  |  | 0.0249 (0.0258) |
| Threat Level (logged) x Category: Research and Public Policy |  |  | -0.0177 (0.0204) |
|  |  |  |  |
| Fixed-Effects: |  |  |  |
| County | Yes | No | No |
| Month-Year | Yes | No | No |
| Individual | No | Yes | Yes |
| Date | No | Yes | Yes |
|  |  |  |  |
| S.E. Clustered by: | State | Individual & State | Individual & State |
| Weights: | County Population | None | None |
|  |  |  |  |
| Observations | 116,480 | 696,942 | 617,657 |
| R2 | 0.82726 | 0.83448 | 0.85416 |
| Within R2 | 0.0000568 | 2.31E-05 | 0.06418 |

**Table S2**. Table shows full regression results of CN models using the log transformed threat measure. The dependent variable for all models was log-transformed donation amounts. Including the category interactions in the individual-level model reduced the number of observations due to missing category labels. Significance codes: *** p < .001, ** p < .01, * p < .05, † p < .1.

**Table S3. Dictator-Game Regression Table – Logged Threat Level.**

| Threat Level (logged) | 0.0705 (0.0429) |
| --- | --- |
|  |  |
| Fixed-Effects: |  |
| Individual | Yes |
| Wave | Yes |
|  |  |
| S.E. Clustered by: | Individual & State |
| Weights: | None |
|  |  |
| Observations | 4,272 |
| R^2^ | 0.72841 |
| Within R^2^ | 0.00114 |

**Table S3**. Table shows full regression results of the dictator-game model using the log transformed threat measure. The dependent variable was the allocation amount. Significance codes: *** p < .001, ** p < .01, * p < .05.

**Table S4. Charity Navigator Regression Table.**

|  | **County-Month Aggregation** | **Individual-**  **Level** | **Individual-**  **Level** |
| --- | --- | --- | --- |
|  |  |  |  |
| Threat Level: Low | 0.3163*** (0.0641) | 0.0275† (0.0145) |  |
| Threat Level: Medium | 0.2849*** (0.0738) | 0.0128 (0.0135) |  |
| Threat Level: High | 0.3294*** (0.0540) | 0.0339* (0.0161) |  |
| Category: Arts, Culture, Humanities |  |  | -0.1433*** (0.0124) |
| Category: Community Development |  |  | 0.1882*** (0.0083) |
| Category: Education |  |  | 0.0168* (0.0079) |
| Category: Environment |  |  | 0.0510*** (0.0045) |
| Category: Health |  |  | 0.1159*** (0.0049) |
| Category: Human and Civil Rights |  |  | 0.0694*** (0.0064) |
| Category: Human Services |  |  | 0.1334*** (0.0050) |
| Category: International |  |  | 0.1924*** (0.0053) |
| Category: Religion |  |  | 0.0695** (0.0215) |
| Category: Research and Public Policy |  |  | -0.6568*** (0.0218) |
| Threat Level: Low x Category: Animals |  |  | -0.0087 (0.0199) |
| Threat Level: Medium x Category: Animals |  |  | -0.0117 (0.0178) |
| Threat Level: High x Category: Animals |  |  | 0.0088 (0.0238) |
| Threat Level: Low x Category: Arts, Culture, Humanities |  |  | 0.0015 (0.0257) |
| Threat Level: Medium x Category: Arts, Culture, Humanities |  |  | -0.0159 (0.0234) |
| Threat Level: High x Category: Arts, Culture, Humanities |  |  | 0.1020** (0.0326) |
| Threat Level: Low x Category: Community Development |  |  | 0.0346 (0.0309) |
| Threat Level: Medium x Category: Community Development |  |  | 0.0165 (0.0240) |
| Threat Level: High x Category: Community Development |  |  | 0.0149 (0.0295) |
| Threat Level: Low x Category: Education |  |  | 0.0554† (0.0330) |
| Threat Level: Medium x Category: Education |  |  | 0.0168 (0.0320) |
| Threat Level: High x Category: Education |  |  | 0.0433 (0.0304) |
| Threat Level: Low x Category: Environment |  |  | -0.0318 (0.0236) |
| Threat Level: Medium x Category: Environment |  |  | -0.0066 (0.0222) |
| Threat Level: High x Category: Environment |  |  | 0.0011 (0.0241) |
| Threat Level: Low x Category: Health |  |  | 0.0005 (0.0221) |
| Threat Level: Medium x Category: Health |  |  | -0.0262† (0.0154) |
| Threat Level: High x Category: Health |  |  | -0.0038 (0.0231) |
| Threat Level: Low x Category: Human and Civil Rights |  |  | 0.0261 (0.0211) |
| Threat Level: Medium x Category: Human and Civil Rights |  |  | 0.0020 (0.0154) |
| Threat Level: High x Category: Human and Civil Rights |  |  | 0.0529* (0.0234) |
| Threat Level: Low x Category: Human Services |  |  | 0.0843*** (0.0188) |
| Threat Level: Medium x Category: Human Services |  |  | 0.0670*** (0.0150) |
| Threat Level: High x Category: Human Services |  |  | 0.0803*** (0.0149) |
| Threat Level: Low x Category: International |  |  | 0.0067 (0.0163) |
| Threat Level: Medium x Category: International |  |  | 0.0053 (0.0126) |
| Threat Level: High x Category: International |  |  | -0.0055 (0.0182) |
| Threat Level: Low x Category: Religion |  |  | 0.0442 (0.0529) |
| Threat Level: Medium x Category: Religion |  |  | 0.0286 (0.0582) |
| Threat Level: High x Category: Religion |  |  | 0.0797 (0.0636) |
| Threat Level: Low x Category: Research and Public Policy |  |  | -0.0194 (0.0435) |
| Threat Level: Medium x Category: Research and Public Policy |  |  | 0.0017 (0.0444) |
| Threat Level: High x Category: Research and Public Policy |  |  | -0.0289 (0.0503) |
|  |  |  |  |
| Fixed-Effects: |  |  |  |
| County | Yes | No | No |
| Month-Year | Yes | No | No |
| Individual | No | Yes | Yes |
| Date | No | Yes | Yes |
|  |  |  |  |
| S.E. Clustered by: | State | Individual & State | Individual & State |
| Weights: | County Population | None | None |
|  |  |  |  |
| Observations | 116,480 | 696,942 | 617,657 |
| R^2^ | 0.82735 | 0.83448 | 0.8542 |
| Within R^2^ | 0.00063 | 0.0000554 | 0.06446 |

**Table S4**. Table shows full regression results of CN models described in the text. The dependent variable for all models was log-transformed donation amounts. Including the category interactions in the individual-level model reduced the number of observations due to missing category labels. Significance codes: *** p < .001, ** p < .01, * p < .05, † p < .1.

**Table S5. Charity Navigator County-Level Models, Median Household Income (MHI) Interactions.**

|  | **Continuous**  **MHI** | **Median Split**  **MHI** | **Continuous MHI** | **Median Split MHI** |
| --- | --- | --- | --- | --- |
|  |  |  |  |  |
| Threat Level: Low | 0.1840† (0.0939) | 0.2649*** (0.0619) |  |  |
| Threat Level: Medium | -0.1063 (0.1641) | 0.2221** (0.0799) |  |  |
| Threat Level: High | 0.2021 (0.1552) | 0.3180*** (0.0578) |  |  |
| Threat Level: Low x MHI | 1.77e-6 (1.58e-6) |  |  |  |
| Threat Level: Medium x MHI | 5.72e-6* (2.18e-6) |  |  |  |
| Threat Level: High x MHI | 1.92e-6 (2.43e-6) |  |  |  |
| Threat Level: Low x Above Median MHI |  | 0.0847 (0.0762) |  |  |
| Threat Level: Medium x Above Median MHI |  | 0.1242 (0.0788) |  |  |
| Threat Level: High x Above Median MHI |  | 0.0239 (0.0714) |  |  |
| Threat Level (logged) |  |  | -0.0803 (0.0667) | 0.0134 (0.0261) |
| Threat Level (logged) x MHI |  |  | 1.78e-6† (9.37e-7) |  |
| Threat Level (logged) x Above Median MHI |  |  |  | 0.0500 (0.0308) |
|  |  |  |  |  |
| Fixed-Effects: |  |  |  |  |
| County | Yes | Yes | Yes | Yes |
| Month-Year | Yes | Yes | Yes | Yes |
|  |  |  |  |  |
| S.E. Clustered by: | State | State | State | State |
| Weights: | County Population | County Population | County Population | County Population |
|  |  |  |  |  |
| Observations | 115,492 | 115,492 | 115,492 | 115,492 |
| R^2^ | 0.82653 | 0.82652 | 0.82642 | 0.82641 |
| Within R^2^ | 0.00082 | 0.00072 | 0.00016 | 0.00012 |

**Table S5**. Table shows regression results using county-level CN data, which include interactions between threat level and county-level median household income (MHI). Threat level is categorical in the left two columns, and log-transformed in the right two. MHI was included either as a continuous variable (labeled “MHI”), or median split (1 = county MHI is greater than or equal to the U.S.-wide MHI, 0 = otherwise, labeled “Above Median MHI”). Interactions with log-transformed MHI obtained similar results. Significance codes: *** p < .001, ** p < .01, * p < .05, † p < .1.

**Table S6. Dictator-Game Regression Table.**

| Threat Level: Low | 0.2502* (0.0968) |
| --- | --- |
| Threat Level: Medium | 0.3813** (0.1106) |
| Threat Level: High | 0.2417* (0.0946) |
|  |  |
| Fixed-Effects: |  |
| Individual | Yes |
| Wave | Yes |
|  |  |
| S.E. Clustered by: | Individual & State |
| Weights: | None |
|  |  |
| Observations | 4,272 |
| R^2^ | 0.72942 |
| Within R^2^ | 0.00482 |

**Table S6**. Table shows full regression results of the dictator-game model described in the text. The dependent variable was the allocation amount. Significance codes: *** p < .001, ** p < .01, * p < .05.

**SI References**

1. Anonymous (2019) U.S. Census Bureau QuickFacts: United States.
